# Supplementary material for: Reconciling Mining with the Conservation of Cave Biodiversity: A Quantitative Baseline to Help Establish Conservation Priorities
Source: PLoS One. 2016 Dec 20;11(12):e0168348. doi: 10.1371/journal.pone.0168348 (PMC5173368; doi:10.1371/journal.pone.0168348)
Supplement: S1 Dataset — (ZIP) [file pone.0168348.s002.zip › Taxa/Serra Norte/SN_2013/N4E/Lista N4E-90.pdf]

## CAVIDADE N4E-0090

| Classe | Ordem | Fam/Outros | Gên/Outros | Espécie | S-U | U-S | Zon. |
|---|---|---|---|---|---|---|---|
| Arachnida | Araneae | Ochyroceratidae | *Speocera* | sp.1 | | X | E |
| Arachnida | Araneae | Pholcidae | *Mesabolivar* | sp.1 | X | X | E |
| Arachnida | Araneae | Salticidae | | sp.4 | X | | E |
| Arachnida | Araneae | Salticidae | | sp.8 | X | | E |
| Arachnida | Araneae | Theridiidae | | jovem | | X | E |
| Arachnida | Araneae | Theridiosomatidae | *Plato* | sp.1 | X | X | E |
| Arachnida | Opiliones | Cosmetidae | *Roquettea* | *singularis* | 1 | | E |
| Arachnida | Opiliones | Stygnidae | *Protimesius* | *gracilis* | 2 | | E |
| Arachnida | Pseudoscorpiones | Chernetidae | *Spelaeochernes* | sp.1 | X | X | E |
| Entognatha | Collembola | Paronellidae | | sp.1 | | X | E |
| Insecta | Coleoptera | Curculionidae | | sp.2 | X | | E |
| Insecta | Coleoptera | Staphylinidae | Pselaphinae | sp.21 | X | | E |
| Insecta | Coleoptera | Staphylinidae | Pselaphinae | sp.37 | X | | E |
| Insecta | Coleoptera | Staphylinidae | | sp.44 | X | | E |
| Insecta | Coleoptera | Staphylinidae | | sp.45 | X | | E |
| Insecta | Diptera | Chironomidae | | sp. | X | | E |
| Insecta | Diptera | Culicidae | *Culex* | sp. | X | | E |
| Insecta | Diptera | Drosophilidae | | sp. | X | | E |
| Insecta | Diptera | Sciaridae | | sp. | X | | E |
| Insecta | Diptera | Tipulidae | Tipulinae | sp. | X | | E |
| Insecta | Heteroptera | Cydnidae | | sp. | X | X | E |
| Insecta | Homoptera | Cixiidae | | jovem | X | | E |
| Insecta | Hymenoptera | Formicidae | *Camponotus* | sp.1 | X | | E |
| Insecta | Hymenoptera | Formicidae | *Camponotus* | sp.2 | X | | E |
| Insecta | Hymenoptera | Formicidae | *Pachycondyla* | sp.1 | | X | E |
| Insecta | Hymenoptera | Formicidae | *Pachycondyla* | sp.2 | X | X | E |
| Insecta | Hymenoptera | Formicidae | *Pheidole* | sp.1 | | X | E |
| Insecta | Hymenoptera | Formicidae | *Pheidole* | sp.2 | X | | E |
| Insecta | Isoptera | | | sp. | X | | E |
| Insecta | Lepidoptera | Noctuoidea | | sp.5 | | 1 | E |
| Insecta | Lepidoptera | | | jovem | X | | E |
| Insecta | Orthoptera | Phalangopsidae | *Phalangopsis* | sp. | 1 | 3 | E |
| Insecta | Psocoptera | Psyllipsocidae | | jovem | X | X | E |
| Insecta | Thysanura | Nicoletiidae | | jovem | | X | E |
| Gastropoda | Pulmonata | Bulimulidae | *Naesiotus* | sp. | X | | E |
| Gastropoda | Pulmonata | Systrophiidae | *Happia* | sp. | X | | E |
| Symphyla | | Scutigerellidae | | sp. | X | X | E |
| Turbellaria | Tricladida | | | sp.1 | 1 | | E |
| Mammalia | Chiroptera | Phyllostomidae | *Carollia* | *perspicillata* | | 1 | P |
